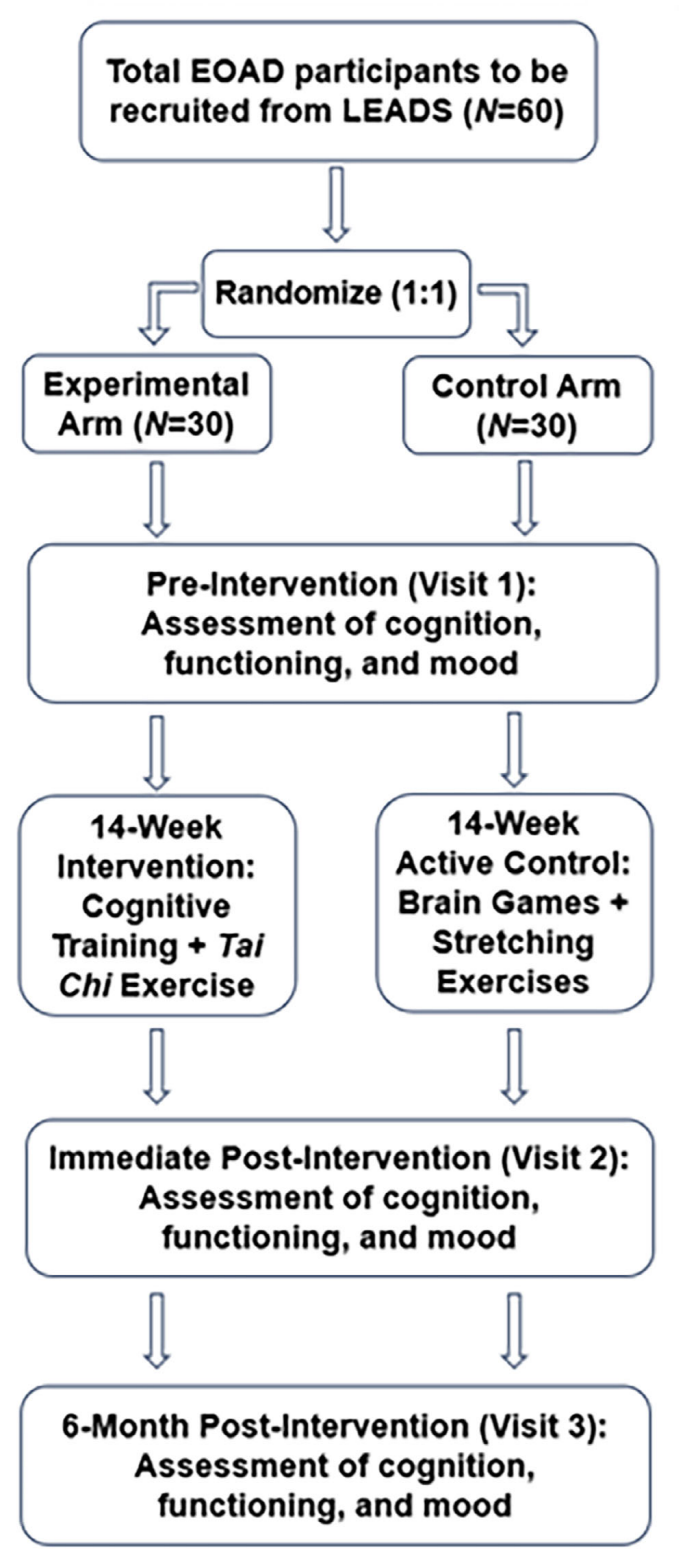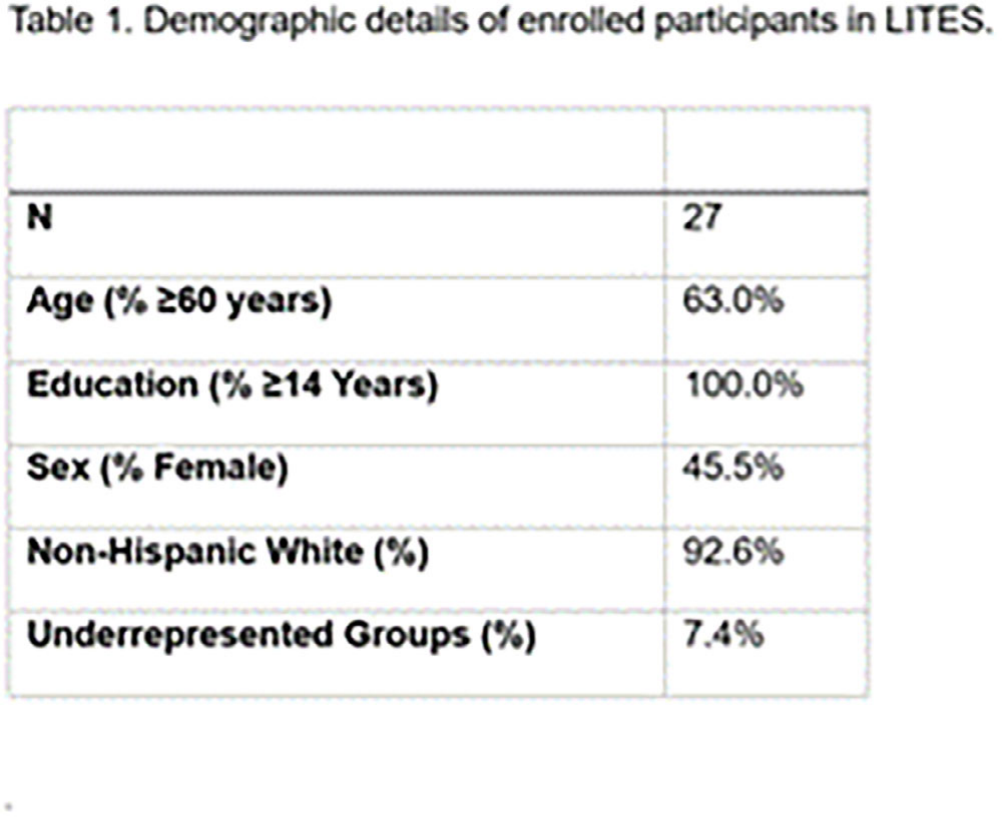# Randomized Combined Lifestyle Intervention for the Treatment of Early‐Onset Alzheimer’s Disease: Design and Preliminary Feasibility Outcomes

**DOI:** 10.1002/alz70861_108864

**Published:** 2025-12-23

**Authors:** Dustin B. Hammers, Jane Musema, Kala Kirby, Amy Trullinger, Amy Pottenger, Frederick W. Unverzagt, Liana G. Apostolova

**Affiliations:** ^1^ Indiana University School of Medicine, Indianapolis, IN USA; ^2^ Department of Neurology, Indiana School of Medicine, Indianapolis, IN USA; ^3^ Indiana University, Indianapolis, IN USA; ^4^ Indiana Alzheimer's Disease Research Center, Indianapolis, IN USA; ^5^ Department of Medical and Molecular Genetics, Indiana University School of Medicine, Indianapolis, IN USA; ^6^ Department of Neurology, Indiana University School of Medicine, Indianapolis, IN USA; ^7^ Department of Radiology and Imaging Sciences, Center for Neuroimaging, Indiana University School of Medicine, Indianapolis, IN USA

## Abstract

**Background:**

Despite documented benefit in cognitively normal populations, combined cognitive training and physical exercise interventions have had limited consideration in Alzheimer’s disease cohorts. This is particularly true in Early‐Onset Alzheimer’s Disease (EOAD) samples, as the rareness of the condition has to date limited the examination of either pharmacologic or behavioral interventions. The Lifestyle Interventions for the Treatment of Early‐Onset Alzheimer’s Disease Study (LITES) seeks to generate preliminary data regarding feasibility and efficacy of a combined cognitive training and *Tai Chi* exercise lifestyle intervention in participants with EOAD.

**Method:**

LITES is an NIA‐funded phase IIb blinded randomized clinical trial recruiting participants with amyloid‐positive EOAD through an ongoing multi‐center study. Following remote baseline evaluation of cognition, functioning, and mood, participants are randomized into (1) Computerized Cognitive Training (using *BrainHQ*) + *Tai Chi* Exercise Training or (2) Active Control (online brain games + stretching). Both arms are asked to complete 14 weeks of intervention (40 hours of cognitive and 14 hours of exercise training). Feasibility and efficacy outcome measures were repeated post‐intervention and 6‐months later. See Figure 1.

**Result:**

LITES has currently enrolled 27 participants across 8 sites, who have been predominantly non‐Hispanic White (92.6%), highly educated (all ≥14 years), and over the age of 60 (63.0%). See Table 1. Gender representation has been equal. Nine participants withdrew from the study, with area deprivation index score displaying a medium effect on withdrawal status (*d*=0.44). Twelve participants have currently completed their assigned intervention program. Of those completers, mean cognitive and exercise training times were 35.6 and 13.0 hours, respectively. Full doses of training were obtained for one‐half of participant for cognitive training, and for two‐thirds of participants for exercise training. All completers found their assigned training program enjoyable and safe to practice, and 83.3% reported their cognitive program moderately‐to‐extremely helpful in improving brain health. Barriers to satisfaction were also identified.

**Conclusion:**

LITES’s current enrollment showcases that EOAD participants found this combined cognitive and exercise intervention feasible and enjoyable. Future results will inform efficacy of this combined lifestyle intervention on short‐term cognition and functioning within EOAD.